# Pelvic and breast examination skills curricula in United States medical schools: a survey of obstetrics and gynecology clerkship directors

**DOI:** 10.1186/s12909-016-0835-6

**Published:** 2016-12-16

**Authors:** Lorraine Dugoff, Archana Pradhan, Petra Casey, John L. Dalrymple, Jodi F. Abbott, Samantha D. Buery-Joyner, Alice Chuang, Amie J. Cullimore, David A. Forstein, Brittany S. Hampton, Joseph M. Kaczmarczyk, Nadine T. Katz, Francis S. Nuthalapaty, Sarah M. Page-Ramsey, Abigail Wolf, Nancy A. Hueppchen

**Affiliations:** 1 University of Pennsylvania Perelman School of Medicine, 3400 Spruce Street, 2 Silverstein Building, Philadelphia, PA 19104 USA; 2 Department of Obstetrics and Gynecology, Robert Wood Johnson Medical School, New Brunswick, NJ USA; 3 Department of Obstetrics and Gynecology, Mayo Clinic, Rochester, MN USA; 4 Department of Obstetrics and Gynecology, Beth Israel Deaconess Medical Center, Harvard Medical School, Boston, MA USA; 5 Department of Obstetrics and Gynecology, Boston University School of Medicine, Needham, MA USA; 6 Department of Obstetrics and Gynecology, Virginia Commonwealth University School of Medicine Inova Campus, Falls-Church, VA USA; 7 Department of Obstetrics and Gynecology, University of North Carolina School of Medicine, Chapel Hill, NC USA; 8 Department of Obstetrics and Gynecology, McMaster University, Hamilton, ON Canada; 9 Department of Obstetrics and Gynecology, Greenville Health System University Medical Center, Greenville, SC USA; 10 Department of Obstetrics and Gynecology, Brown Alpert Medical School, Providence, RI USA; 11 Department of Obstetrics and Gynecology, Philadelphia College of Osteopathic Medicine, Philadelphia, PA USA; 12 Department of Obstetrics & Gynecology and Women’s Health, Albert Einstein College of Medicine, Bronx, NY USA; 13 Department of Obstetrics and Gynecology, Greenville Health System University Medical Center, Greenville, SC USA; 14 Department of Obstetrics and Gynecology, University of Texas Health Science Center, San Antonio, TX USA; 15 Department of Obstetrics and Gynecology, Jefferson Medical College, Philadelphia, PA USA; 16 Department of Obstetrics and Gynecology, Johns Hopkins Medical Institutions, Baltimore, MD USA

**Keywords:** Pelvic examination, Breast examination, Obstetrics and gynecology clerkship, Undergraduate medical education, Medical students

## Abstract

**Background:**

Learning to perform pelvic and breast examinations produces anxiety for many medical students. Clerkship directors have long sought strategies to help students become comfortable with the sensitive nature of these examinations. Incorporating standardized patients, simulation and gynecologic teaching associates (GTAs) are approaches gaining widespread use. However, there is a paucity of literature guiding optimal approach and timing. Our primary objective was to survey obstetrics and gynecology (Ob/Gyn) clerkship directors regarding timing and methods for teaching and assessment of pelvic and breast examination skills in United States medical school curricula, and to assess clerkship director satisfaction with current educational strategies at their institutions.

**Methods:**

Ob/Gyn clerkship directors from all 135 Liaison Committee on Medical Education accredited allopathic United States medical schools were invited to complete an anonymous 15-item web-based questionnaire.

**Results:**

The response rate was 70%. Pelvic and breast examinations are most commonly taught during the second and third years of medical school. Pelvic examinations are primarily taught during the Ob/Gyn and Family Medicine (FM) clerkships, while breast examinations are taught during the Ob/Gyn, Surgery and FM clerkships. GTAs teach pelvic and breast examinations at 72 and 65% of schools, respectively. Over 60% of schools use some type of simulation to teach examination skills. Direct observation by Ob/Gyn faculty is used to evaluate pelvic exam skills at 87% of schools and breast exam skills at 80% of schools. Only 40% of Ob/Gyn clerkship directors rated pelvic examination training as excellent, while 18% rated breast examination training as excellent.

**Conclusions:**

Pelvic and breast examinations are most commonly taught during the Ob/Gyn clerkship using GTAs, simulation trainers and clinical patients, and are assessed by direct faculty observation during the Ob/Gyn clerkship. While the majority of Ob/Gyn clerkship directors were not highly satisfied with either pelvic or breast examination training programs, they were less likely to describe their breast examination training programs as excellent as compared to pelvic examination training—overall suggesting an opportunity for improvement. The survey results will be useful in identifying future challenges in teaching such skills in a cost-effective manner.

**Electronic supplementary material:**

The online version of this article (doi:10.1186/s12909-016-0835-6) contains supplementary material, which is available to authorized users.

## Background

Teaching pelvic and breast examination skills is an integral component of clinical medical education. Due to the sensitive nature of these examinations, medical students may find these examinations particularly challenging and awkward. Unlike many skills routinely learned on other clinical clerkships, students may be uncomfortable and hesitant to perform pelvic and breast examinations on actual patients [1–3]. This issue has been shown to be particularly true for male medical students and students from cultures with strong taboos against interpersonal physical contact [1]. Gynecologic teaching associates (GTAs), standardized patients (SPs) and simulation including the use of pelvic trainers have been used to help students learn in a more comfortable and supportive setting, though the best practice has yet to be defined. Furthermore, the optimal point in the medical curriculum at which time these skills should be taught has not been established. In many schools the skills are taught as part of a clinical rotation that could occur at any point in the second or third year, while at others the skills are taught at specific times in the curriculum unrelated to the clinical rotations.

In order to inform curricular enhancement and potential future educational research efforts, we surveyed obstetrics and gynecology (Ob/Gyn) clerkship directors throughout the country about their pelvic and breast examination teaching practices. We sought to determine current timing and strategies used for teaching these skills in United States medical school curricula along with an assessment of clerkship director satisfaction with the current teaching process.

## Methods

Obstetrics and gynecology clerkship directors from all 135 Liaison Committee on Medical Education (LCME) accredited allopathic United States medical schools were invited to complete an anonymous 15-item web-based questionnaire using SurveyMonkey (Palo Alto, CA) (Additional file 1). The survey questions related to timing and method of teaching pelvic and breast examination skills. In survey question number seven, “other live model” refers to a standardized patient acting as a live patient with a faculty instructor. Clerkship directors who rated their pelvic and/or breast examination skills curricula as adequate or fair were asked what they would like to modify.

Two invitations to participate were sent by email from the Association of Professors of Gynecology and Obstetrics (APGO) on behalf of the Undergraduate Medical Education Committee by e-mail over a two-week interval in July 2013. An exemption for this project was obtained from the University of Pennsylvania Institutional Review Board. No incentive was provided for participation.

The questionnaire was developed by authors LD, AP and NH, and reviewed for content validity, overall design, and usability by the APGO Undergraduate Medical Education Committee. Feedback was incorporated and the questionnaire was approved by all authors. The survey was pilot-tested by the APGO Undergraduate Medical Education Committee. Survey pilot results were analyzed for nonresponses, missing data, and comments by respondents, which led to additional revisions to complete the internal validation process. Anonymous survey results were analyzed using standard descriptive statistics (Microsoft Excel 2010).

## Results

Ninety-five of 135 clerkship directors completed the survey, yielding a 70% response rate. Pelvic and breast examination skills are taught during a patient communication and physical examination or transition to clinical clerkships course within the pre-clinical years at 89% (*n* = 85) and 86% (*n* = 82) of medical schools, respectively. Seventeen percent of schools (*n* = 16) offer pelvic examination skill training during the first pre-clinical year, 81% (*n* = 77) offer it during the second year, and 8% of schools (*n* = 8) provide training during the first and second years. Fourteen percent of schools (*n* = 13) offer breast examination skill training during the first pre-clinical year, 80% (*n* = 76) offer it during the second year, and 7% (*n* = 7) provide training during the first and second years. Eleven percent (*n* = 10) and 14% of schools (*n* = 13) do not teach pelvic and breast examination skills, respectively, prior to clinical clerkships.

All Ob/Gyn directors who responded provide training in pelvic examination skills and 88% (*n* = 84) of the Ob/Gyn clerkships provide training in breast examination skills. Teaching of pelvic and breast examination skills on the Ob/Gyn clerkship is primarily done by health care providers, both faculty and residents. Certified nurse midwives, nurse practitioners and physician assistants have a significant role in teaching pelvic and breast examinations at 33% (*n* = 31) and 22% (*n* = 21) of schools, respectively. Pelvic examination skills are taught on the family medicine (FM) and internal medicine (IM) clerkships at 34% (*n* = 32) and 1% (*n* = 1) of the medical schools, respectively, while breast examination skills are taught on 41% (*n* = 39) of surgery, 28% (*n* = 27) of FM and 5% (*n* = 5) of IM clerkships. Five percent (*n* = 5) of schools offer longitudinal integrated curricula which include instruction in both pelvic and breast examination skills.

**Fig. 1:**
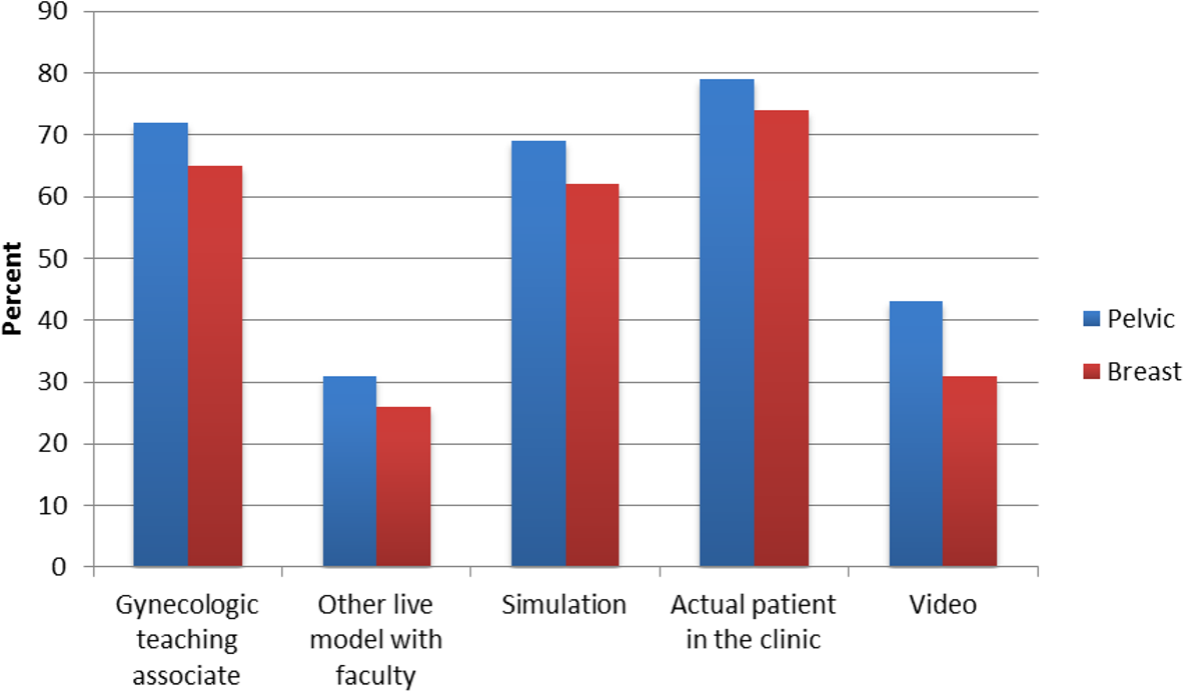
Current distribution of methods used to teach pelvic and breast skills

Most clerkship directors reported that they use multiple methods to teach pelvic and breast examination skills (Fig. 1). GTAs are utilized to teach pelvic and breast examination skills at 72% (*n* = 68) and 65% (*n* = 62) of schools, respectively. Over 60% of the schools use simulation to teach the examination skills.

**Fig. 2:**
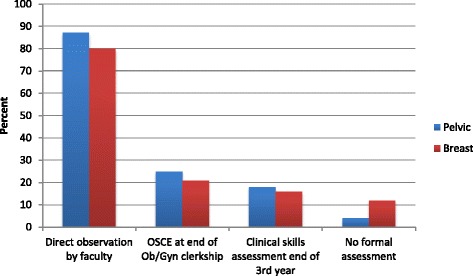
Approach used to formally assess pelvic and breast exam skills

The majority of Ob/Gyn clerkships have faculty directly observe and evaluate students (Fig. 2). An objective structured clinical exam (OSCE) is used to assess pelvic examination skills at the end of the Ob/Gyn clerkship at 25% (*n* = 24) of the schools while 18% (*n* = 15) of schools evaluate pelvic examination skills as part of a clinical skills examination at the end of the third year. Corresponding values for the breast skills evaluation using an OSCE at the end of the clerkship and a clinical skills examination at the end of the third year are 21% (*n* = 20) and 16% (*n* = 15), respectively.

Of Ob/Gyn clerkship directors who responded, 20% (*n* = 19) did not require a specific number of pelvic examinations and 31% (*n* = 29) did not require a specific number of breast examinations to be performed over the course of the clerkship. Of the clerkships with a defined required minimum number of pelvic and breast exams, the range was 1 to 10 with means of 2.84 ± 2.29 and 2.33 ± 1.85, respectively.

**Fig. 3:**
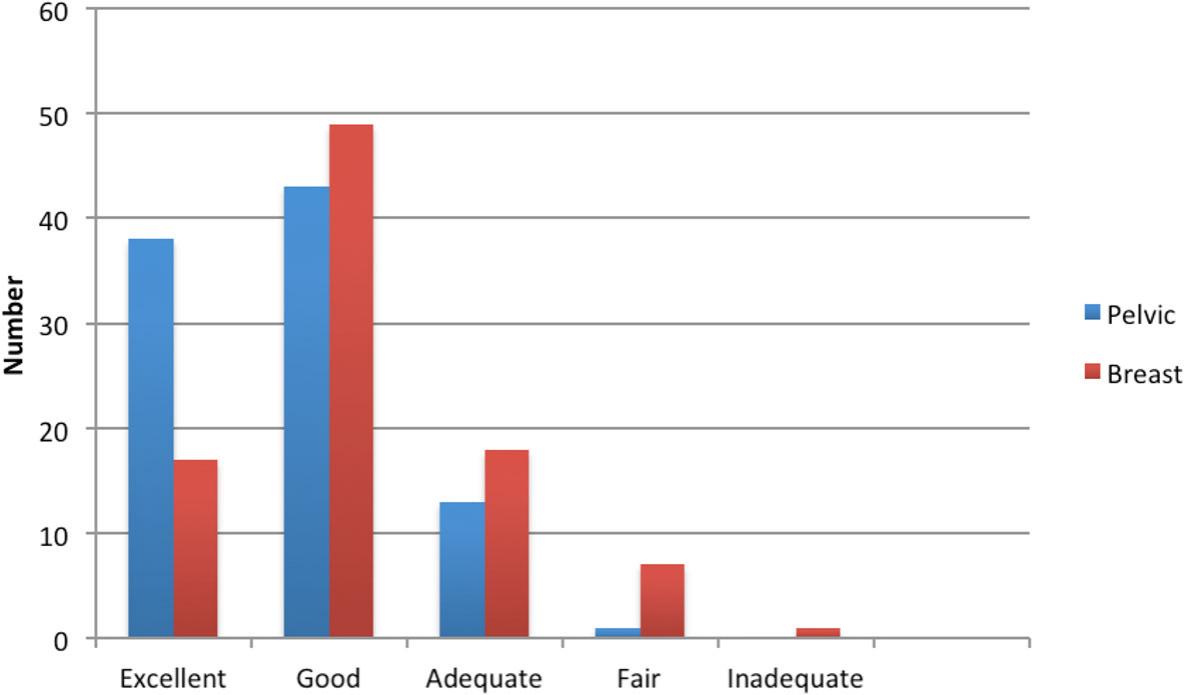
Ob/Gyn clerkship director rating of pelvic and breast exam skills curricula

Obstetrics and gynecology clerkship directors were more likely to rate the pelvic examination training curriculum at their school as excellent (40%) compared to the breast examination training curriculum (18%). Approximately twice as many clerkship directors rated the breast examination curriculum fair or adequate (27%) as compared to the pelvic examination curriculum (15%) (Fig. 3). No clerkship director rated the pelvic examination skills curriculum as inadequate, while one clerkship director rated the breast examination curriculum as inadequate. Table 1 outlines the modifications in the curriculum suggested by clerkship directors who rated their pelvic and breast skills programs as fair or inadequate.

**Table 1:** Clerkship director suggested modifications to pelvic and breast skills training

Pelvic Exam Training Fair or Inadequate	
Respondent suggested modifications: • Additional longitudinal teaching and assessment	
Breast Exam Training Fair or Inadequate	
Respondent suggested modifications • Increased instruction • Live models or standardized patients • Use GTAs for teaching examination • Development of more clearly defined interdisciplinary curriculum and assessment • Covered best in surgery clerkship where more time devoted • Teach shorter, more practical exam sequence	

## Discussion

There is scant literature regarding the optimal timing or approach for teaching and assessment of pelvic and breast examination skills. A monograph on Recommendations for Clinical Skills Curricula published by the Association of American Medical Colleges (AAMC) in 2005 acknowledged that generally accepted standards regarding what skills should be learned and how well they can be performed during the undergraduate medical experience do not currently exist and that medical schools vary widely in the degree to which they specify skills expectations for students [4]. This monograph noted that the Association of Professors in Obstetrics and Gynecology (APGO), the Association for Surgical Education (ASE), the Clerkship Directors in Internal Medicine (CDIM) and the Society of Teachers of Family Medicine (STFM) uniformly recommend that all learners reach competency in pelvic examination skills while APGO, CDIM and the Council on Medical Student Education in Pediatrics (ComSEP) all endorsed undergraduate medical education curricula for the breast examination [4].

In 2008 the AAMC convened the Preclerkship Clinical Skills Education Task Force in order to establish a national consensus regarding preclinical skills. The task force recommended that students should enter clerkships at the advanced beginner level for performing a pelvic and breast examination as well as for describing the examination process and findings. They further recommended that various formats should be used to facilitate preclerkship clinical skills acquisition from physician and non-physician faculty. The task force acknowledged simulation as optimal for the initial clinical skills acquisition [5].

Our national survey of Ob/Gyn clerkship directors revealed that pelvic and breast examinations are most commonly taught as part of a preclerkship physical diagnosis course and during the Ob/Gyn clerkship using GTAs, simulation and actual patients. Use of standardized patients, simulation, and actual patients require the time and attention of a faculty member, whereas GTAs combine the role of patient and teacher. Most assessment methods involve direct observation by faculty during the Ob/Gyn clerkship.

Of note, the finding of 14% of the schools that teach the pelvic and breast examination skills in the preclerkship years at their schools is not consistent with the recommendations of the AAMC Preclerkship Clinical Skills Education Task Force. While imaging may have largely replaced detailed pelvic and breast examinations, the performance of a pelvic and breast examination is still an important skill for all specialties caring for women. The undifferentiated medical student in various settings, initially assesses a patient presenting with a breast complaint, pelvic pain, vaginal bleeding or a pelvic mass. The initial examination gives the provider information necessary for a more directed referral for imaging or specialty care.

Forty percent of clerkship directors rated their pelvic examination curriculum as excellent whereas only 18% rated their breast examination curriculum as excellent. This presents opportunities for improvement in this regard. Given the breast examination is taught with great variability across many disciplines, this finding is understandable. We speculate that having a specific discipline take ownership of developing and promulgating a standardized breast examination curriculum, much like the pelvic examination is primarily “owned” by Ob/Gyn, would likely improve satisfaction. Currently it is difficult to assess competency in the breast examination without a standard curriculum.

Ob/Gyn clerkships where learning and assessment of pelvic examination skills have traditionally been concentrated vary in length. Remmen et al. reported that clinical clerkship experiences alone were insufficient to provide adequate training in basic clinical skills including gynecologic and obstetric skills at three European medical schools [6]. The authors did not specify whether the sites provided any initial GTA or other training at the beginning of the clerkship and did not address the optimal timing for pelvic examination training. If disciplines coordinated efforts to create a developmental and longitudinal approach to teaching these skills, the exposure in a single clerkship would be less important and reinforcement by repetition could be easily accomplished. To fully answer our question regarding optimal skills training, we also need to study learner outcomes, and whether assessment methods are comparable in measuring competency following various skills training programs.

Several methods for teaching pelvic and breast examination skills have been reported in the literature including didactic lectures or videos, standardized patients, GTAs and faculty instruction using simulation and actual patients in the clinical setting [1]. A number of published studies have demonstrated the effectiveness of GTAs in teaching method [7–11] and assessment [12]. One advantage of the GTA model is the opportunity for immediate feedback and for formative assessment. The GTA method may be cost-effective as compared to faculty teaching. Pradhan and colleagues randomized 106 medical students at the same obstetrics and gynecology clerkship clinical site to receive pelvic examination training either with a GTA or a SP and an obstetrics and gynecology faculty member [13]. All of the students received a 1-h lecture on pelvic examination skills prior to the training. There was no significant difference in gynecologic examination scores on an OSCE at the end of the clerkship. Students in both groups felt more confident after training and found the sessions valuable. The GTA training approach, obviating the need for additional faculty, was associated with a cost-savings. The authors concluded that faculty time and effort need not be utilized for pelvic examination training.

Technology-enhanced simulation, defined as an educational tool or device with which the learner physically interacts to mimic an aspect of clinical care, is associated with statistically significant improvements in learner outcomes in teaching pelvic and breast examination skills [14]. A comprehensive systematic review and meta-analysis concluded that interventions that allow for greater feedback led to better outcomes [14]. Studies indicate that learners acquire greater proficiency in pelvic examination skills when SPs are added to the simulation model and when models with enhanced feedback are used. Learners in the breast examination studies acquired more skills from a model with tactile feedback or enhanced variability between trials. The authors of the systematic review concluded that SP enhanced curriculum adds beyond that of technology-enhanced simulation alone [14].

A combination of SPs, GTAs and technology-enhanced simulation may be the optimal approach to pelvic and breast examination training. While SPs and GTAs have the advantage of live interaction and feedback, they are typically limited to normal findings whereas simulation may enrich the curriculum with abnormal findings [14]. Further research is needed to determine the optimal approach for incorporating simulators into pelvic and breast examination training. Technology-enhanced simulation may increase clerkship director ratings of pelvic and breast examination training and ability of medical schools to assess these skills at other points in the medical school curriculum.

An additional resource for teaching pelvic and breast examination skills is the APGO Clinical Skills Curriculum Teaching Modules which may be accessed through the APGO website (http://www.apgo.org) under the Basic Clinical Skills heading in the Educational Resources section. Learning outcomes, best practices, a checklist and a performance assessment are provided for each exam skill. The modules may be useful for educators as resources for curriculum development. They may be utilized for teaching and assessment in a simulation or clinical setting in a more standardized fashion.

Our study is limited by the 70% response rate. It is possible that the results of our survey are skewed due to selection bias. All clerkship directors at LCME-accredited medical schools did not respond and thus the data may not be representative of all schools. In addition, the data may be limited by recall bias and accuracy of reporting. In particular, the Ob/Gyn clerkship directors may not have accurate data regarding the pelvic and breast exam skills teaching and curriculum on the internal medicine, surgery and family medicine clerkships. While our survey assessed clerkship director satisfaction with the pelvic and breast examination skill curricula, we did not investigate why clerkship directors were or were not satisfied or how to improve clerkship director satisfaction. Lastly, due to the anonymous nature of the survey, we were unable to attribute specific qualities and characteristics to the most highly rated and the lowest rated training programs.

The data derived from our survey provide observations we can use to devise specific and focused hypothesis driven studies leading to improvement in educational curricula. Future areas of study will focus on optimal timing of teaching pelvic and breast examination skills within the curriculum, the most effective and cost-effective teaching and assessment approaches, the optimal and minimum number of examinations for skill mastery, and student satisfaction and comfort associated with various teaching modalities. Future investigation may also focus on the impact of the pelvic and breast examination curricula on student competence in performing the pelvic and breast examinations, overall satisfaction with the obstetrics and gynecology clerkship, clerkship performance, and long-term interest in pursuing a career in women’s health including obstetrics and gynecology.

## Conclusions

Although the AAMC Pre-clerkship Clinical Skills Education Task Force recommended that students should enter clerkships at the advanced beginner level for performing a pelvic and breast examination, there is scant literature regarding the optimal timing or approach for teaching and assessment of pelvic and breast examination skills. Our survey showed that pelvic and breast examinations are most commonly taught during the Ob/Gyn clerkship using GTAs, simulation trainers and clinical patients, and are assessed by direct faculty observation during the Ob/Gyn clerkship. While the majority of Ob/Gyn clerkship directors were not highly satisfied with either pelvic or breast examination training programs, they were less likely to describe their breast examination training programs as excellent as compared to pelvic examination training—overall suggesting an opportunity for improvement.

## Additional file

Additional file 1: Survey. (DOCX 20 kb)

## Abbreviations

AAMC: Association of American Medical Colleges
APGO: Association of Professors of Gynecology and Obstetrics
CDIM: Clerkship Directors in Internal Medicine
FM: Family medicine
GTAs: Gynecologic teaching associates
IM: Internal medicine
LCME: Liaison Committee on Medical Education
Ob/Gyn: Obstetrics and gynecology
OSCE: Objective structured clinical exam
SPs: Standardized patients
