# Supplementary material for: Pelvic and breast examination skills curricula in United States medical schools: a survey of obstetrics and gynecology clerkship directors
Source: BMC Med Educ. 2016 Dec 16;16:314. doi: 10.1186/s12909-016-0835-6 (PMC5162080; doi:10.1186/s12909-016-0835-6)
Supplement: Additional file 1: — Survey. (DOCX 20 kb) [file 12909_2016_835_MOESM1_ESM.docx]

Supplemental file 1. Survey

Question 1

When are BREAST examinations taught in your curriculum? If the skills are taught at multiple times, please choose all that apply.

- Preclinical year 1
- Preclinical year 2
- Core clerkship year
- I don’t know
- Other (please specify)

Question 2

During which course/clerkships are BREAST examinations taught in your curriculum? If the skills are taught at multiple times, please choose all that apply.

- Basic science course
- Patient communication and physical exam course
- Transition to clinical clerkship year course (Patient communication and physical exam course)
- Ob/Gyn Clerkship
- Internal Medicine Clerkship
- Family Medicine Clerkship
- Surgery Clerkship
- Longitudinal Integrated Clerkship
- I don’t know
- Other (please specify)

Question 3

More specifically, who teaches the BREAST exam? Choose all that apply.

- Ob/Gyn clerkship director
- Other Ob/Gyn faculty
- Surgery clerkship director
- Surgery faculty
- Internal medicine clerkship director
- Internal medicine faculty
- Family medicine clerkship director
- Family medicine faculty
- GTAs (Gynecologic Teaching Associate – a woman who utilizes her own body as a teaching tool as she instructs learners on the exam techniques of the breast and pelvic exam)
- CNMs, NPs, Pas
- Ob/Gyn residents
- Other residents
- Other (please specify)

Question 4

When are PELVIC examinations taught in your curriculum? If the skills are taught at multiple times, please choose all that apply.

- Preclinical year 1
- Preclinical year 2
- Core clerkship year
- I don’t know

Question 5

During which course/clerkships are PELVIC examinations taught in your curriculum? If the skills are taught at multiple times, please choose all that apply.

- Basic science course
- Patient communication and physical exam course
- Transition to clinical clerkship year course (Patient communication and physical exam course)
- Ob/Gyn Clerkship
- Internal Medicine Clerkship
- Family Medicine Clerkship
- Surgery Clerkship
- Longitudinal Integrated Clerkship
- I don’t know
- Other (please specify)

Question 6

More specifically, who teaches the PELVIC exam? Choose all that apply.

- Ob/Gyn clerkship director
- Other Ob/Gyn faculty
- Internal medicine clerkship director
- Internal medicine faculty
- Family medicine clerkship director
- Family medicine faculty
- GTAs (Gynecologic Teaching Associate – a woman who utilizes her own body as a teaching tool as she instructs leasrners on the exam techniques of the breast and pelvic exam)
- CNMs, NPs, Pas
- Ob/Gyn residents
- Other residents
- Other (please specify)

Question 7

Which methods are used to teach the breast and pelvic exams? Choose all that apply.

- GTA (Gynecologic Teaching Associate – a woman who utilizes her own body as a teaching tool as she instructs leasrners on the exam techniques of the breast and pelvic exam)
- Other live model with faculty teaching the examination skills on a live patient model
- Teaching on a simulator trainer (non-live patient)
- Teaching on a patient
- Video
- Other (please specify)

Question 8

How would you rate the adequacy of your current BREAST examination training curriculum?

- Excellent
- Good
- Adequate
- Fair
- Inadequate

Question 9 (Pop up if applicable)

You marked fair or inadequate. What would you like to modify? (Free text response)

Question 10

How would you rate the adequacy of your current PELVIC examination training curriculum?

- Excellent
- Good
- Adequate
- Fair
- Inadequate

Question 11 (Pop up if applicable)

You marked fair or inadequate. What would you like to modify? (Free text response)

Question 12

How are students’ breast and pelvic examination skills formally evaluated in your curriculum? Choose all that apply.

- Verbal feedback
- Written feedback
- Direct observation and evaluation by faculty during the Ob/Gyn clerkship
- OSCE at the end of the Ob/Gyn clerkship
- End of third year clinical skills exam
- Other (please specify)

Question 13

When are formal assessments of breast and pelvic examination skills performed? Choose all that apply.

- Beginning of 2^nd^ year
- Middle of 2^nd^ year
- End of 2^nd^ year
- Beginning of 3^rd^ year
- Beginning of the Ob/Gyn clerkship
- End of the Ob/Gyn clerkship
- End of the 3^rd^ year
- End of the 4^th^ year
- Don’t know
- Other (please specify)

Question 14

How many BREAST exams are students required to perform on the Ob/Gyn clerkship? (Please enter a numerical value greater than or equal to 0.)

Question 15

How many PELVIC exams are students required to perform on the Ob/Gyn clerkship? (Please enter a numerical value greater than or equal to 0.)
